# Twenty-Fourth Annual Session of the Alabama Dental Association

**Published:** 1893-10

**Authors:** 


ARTICLE III.

### TWENTY-FOURTH ANNUAL SESSION OF THE ALABAMA DENTAL ASSOCIATION.

The Alabama Dental Association met in the parlors of the Caldwell Hotel, Birmingham, April 11th, 1893, Dr. C. L. Boyd, Eufaula, President. After an invocation of the Divine blessing on the labors of the Association, by the Rev. T. J. Beard, rector of the Church of the Advent, and the customary addresses of welcome, the President read the annual address.

He spoke of the elevated standing to which the profession of dentistry has attained, and the benefits which it confers on humanity, and dwelt on the unrevealed pain-bililus of preventive dentistry, through the instruction of the people, and especially of mothers, in the comprehension of the demands of nature in the development and preservation of the teeth. He said: We know, or ought to know, the laws of nature and of dental hygiene, and we are untrue to our patrons and to our profession if we fail to impart this knowledge at every opportunity. He spoke of the large number of dentists who, notwithstanding the dental law, and the efforts of the Board of Dental Examiners, are practicing without license—men who are unqualified and unworthy to bear the name of dentist, and urged the setting apart of a fund by the Association for the feeing of lawyers, to see that such persons are brought to justice, and that the law is enforced.

Dr. E. S. Chisholm, Tuscaloosa, spoke of the great difficulty the Board experienced in getting at the facts in regard to parties practicing in violation of the law, and asked the co-operation of all in giving any information they might obtain. Such assistance would be appreciated by those having the subject in hand.

INCIDENTS OF OFFICE PRACTICE

were related and discussed.

Dr. Chisholm spoke of the great advances made in dentistry, especially in saving teeth that formerly would have been condemned. This is a grand move to the front.

Several cases of accidental injuries received, resulting in fractured maxillæ, necrosis, etc., were detailed, and modes of treatment discussed. Also several cases of so called *cancer*, and by proper treatment of oral or rather dental lesions. In one case an alveolar abscess from a lower incisor had been lanced under the chin, near the median line, by a physician who had been treating the patient for several years for cancer.

Dr. A. Eubank, Birmingham, had recently been treating an alveolar abscess, from a devitalized, superior, central incisor, which was discharging opposite the root of the second bicuspid, with a clear tract from one tooth to the other, the incisor being the only dead tooth in the mouth.

Dr. W. G. Browne, Atlanta, Ga., described his method of making a gold partial plate or piece of bridge-work, directly on the plaster model, without the use of dies. The six anterior teeth were the only ones remaining in the upper jaw. They were so badly worn down that Richmond crowns were placed on the four incisors. Gold crowns were placed on the cuspids. Over each of these were telescoped a crown, forming part of a saddle bridge, supporting the bicuspids and molars, from teeth on each side. The two saddles were not connected in the anterior portion of the mouth, but a bar ¼ inch wide, connected the rear ends of the bridges across the roof of the mouth, leaving the rest of the roof uncovered. The entire piece was constructed of No. 30 gage, pure gold, pressed into shape on the plaster model, the bar and saddles were stiffened with a platinum wire soldered to it; enough solder being flowed over to make an even surface. The teeth were put on with rubber attachments; marble dust was mixed with the plaster for the model. The whole piece constituted a double removable bridge, telescoped over the gold cuspid crowns. The teeth on the saddles antagonized with good lower molars, and the bite was raised in crowning the incisors.

Dr. Chisholm renewed his testimony, given at previous meetings, in favor of the Marshall Anchor Denture, which he has used many times with great satisfaction. This plate offers all the advantages of high-priced bridge-work, with almost the cheapness of an ordinary rubber denture.

Dr. R. C. Young, Anniston, related a peculiar case. He had inserted a large contour filling in a central incisor. A year later, the patient (a young boy) returned, with the tooth much elongated and devitalized. He opened from the palatine surface, removed the devitalized pulp, and treated the root canal several times, leaving cotton and carbolic acid in the root canal. After the third sitting the boy failed to return for a year, when he came back for other work, saying that "the front tooth was all right." Having left only carbolized cotton in the root canal, Dr. Young examined the tooth, but failed to find any trace of the opening he had made a year previously. He thought possibly calculus deposits might have filled the opening, but there were absolutely nothing to show that it had ever been drilled.

Dr. T. M. Allen, Birmingham, advocated the use of aristol in place of iodoform in the treatment of root canals.

Dr. H. C. Boyd, Troy, described his method of using the English pinless teeth for rubber work, running gold pins through the holes in the teeth, allowing the ends to project far enough to solder a continuous band to the ends of the pins, inverting in plaster and asbestos for soldering. The teeth are then waxed up and the piece inverted well vulcanized as usual, making a strong but not cumbersome piece.

Dr. T. M. Allen exhibited a set of diagrams, showing teeth with crooked and tortuous root canals, offering for discussion the question:

HOW TO FILL SUCH CANALS.

He also showed a diagram, in colors, of a tooth which had been split open after extraction, showing in the enlarged diagram the very imperfect work in what was supposed to have been a perfectly filled root canal, the colors showing a small portion of chloro-percha near the apex, and a small portion along one side of the root canal—the rest of the canal being unfilled. An interesting discussion followed on the ways and means of treating and filling root canals, the materials employed, the drills and broaches best adapted to the work, what to do with broken off broaches, etc. The method advocated by the majority of the speakers appeared to be as follows: First *find* the canal, with a finely tempered broach; then enlarge it cautiously with graduated Gates-Glidden drills, raising and pushing the drill alternately to be sure it is not caught, depending on the patient to let you know when you have "got there." In case of a broken drill apply pure tincture of iodin and let it rust out; cut away enough of the tooth structure to allow direct access to the root canal for cleaning and filling. Dr. Chisholm advocates cutting "straight through," securing contact of the medicaments with the living tissues outside of the apex, and out through the abscess. In the anterior root of first molars, search carefully for a second canal which is often overlooked and causes subsequent trouble. If the drill unfortunately goes through the side wall, as sometimes happens when the root has an abrupt turn, Dr. C. L. Boyd advises flooding the canal with thin chloro-percha, pumping it in till it goes out through the side. This will close the opening, and not prove an irritant to the external tissues. Dr. Boyd uses a medium size spear point fissure drill to open up the mouth of the canal, then a large size Glidden drill, following down with smaller ones, down to a Donaldson canal broach to reach the apex. Anything that is too minute to be reached by these means does not contain sufficient organic matter to give any trouble.

### FOR FILLING ROOT CANALS.

Dr. A. A. Pearson uses a paste made of oxid of zinc and oil of cloves, into which he works a little of the alloy used in amalgam fillings.

Dr. W. G. Browne uses oxichlorid of zinc because of its mummifying action on any shade of organic matter that may remain. To reduce this possible remainder to the minimum, he uses the hot air cavity dryer to dry the canal out thoroughly. When the bulb is properly treated the copper will be soft enough to be shaped by pressure to the form of the root canal, and he uses it as his root canal filling.

Dr. E. S. Chisholm thinks that if the canal is well cleaned, the apex hermetically sealed, and the opening from the crown cavity also perfectly filled, the space between may be occupied by a column of air with as good results as any more difficult filling; the elements of disease coming from the outside, if both doors are closed access is impossible. For the apex he uses gutta-percha carefully carried down.

Dr. C. L. Boyd thinks that moisture would eventually find its way through cementum and tubuli, and reacting cause gingivitis and pericemental trouble. After thoroughly opening up and cleansing the cavity of debris, using peroxid of hydrogen, Dr. Boyd saturates the canal with carbolic acid, drying out all excess with a little cotton on a broach. He then dips this same moistened cotton in iodoform and dusts the internal walls. The canal is then filled with chloro-percha pumped in. Dr. Boyd believes that with his methods 95 per cent. of all root canals can be opened up to and through the apex, and 90 per cent. can be filled to the apex.

Dr. William Crenshaw, Atlanta, Georgia, uses thin oxichlorid of zinc, imbedding in it a copper or gold wire, after the method of Dr. Morrison, of St. Louis, though preferably he uses a sprig of gutta-percha, avoiding the risk of a fine metal point through the apex. By this method the oxichlorid is driven against the walls of the canal and to the apex by the entrance of the gutta-percha point, and the root canal is well filled.

Dr. T. M. Allen maintained that if all extracted teeth, with roots supposed to be filled, were split open, a large majority would be found to have but very little, if any, filling material in the root canals. It would not be necessary to split open more than three or four without proving this to be the case. He said, "We think we fill them, but all don't do it."

Under the head of

PHYSIOLOGY

the subject of nutrition was discussed at some length.

Dr. C. A. Murill attributes the decay of the teeth of women and children to their large use of starchy foods, as bread and butter, etc. A dentist, to be worthy of the title of doctor, which means *teacher*, should teach his patients about these things, prescribing the proper diet, especially for mothers and children.

Dr. R. R. Freeman, Nashville, Tennessee, spoke at great length on the subject of cleanliness as the great preventive of dental decay. On his bill-heads he inscribes the motto: "Clean teeth don't decay," and if complaint is made of the amount of the bill he refers to the prime cause. He also ascribes much of the trouble with the teeth of young children to modern methods of education. Nature is given no chance—children are forced in the struggle to keep pace with others; to lead a perverted pathological existence; their lives overshadowed by the cloud of miscalled *education*. Instead of being "drawn out" they are deprived and crushed by the burden that is laid on them.

Dr. Chisholm said the good Lord has put an abundance of good things before us, and he made man omnivorous, so we must choose our food from the vegetable and the animal kingdoms. Occupations, climate, modes of life, all have their modifying influence, but too much stress is laid on diet alone.

Dr. Westmoreland, Columbus, Miss., attributes the imperfections in children's teeth to eruptive diseases more than any other factor. With the idea that children "get over" measles, chicken-pox, etc., more easily when very young, they are exposed to the whole list while the teeth are at the most critical stage of formation, and he believes this to be largely the source of the poor teeth.

Dr. A. A. Pearson quoted the case of the Prodigal Son, who lived on husks, but said we had no evidence to show that he had any better teeth than his brothers, who were living on the fat of the land.

Dr. J. Y. Crawford, Nashville, Tenn., attributes the specific affections of the oral cavity to want of functional activity. The peculiar influences of our highly civilized life lessens the functional activity of tooth structure, and results in lesions of the entire organism. The nervous system, the osseous system, the vascular system, are all component parts of the human organism, and all suffer from our present modes of life. We work the brain till it outstrips the body, and especially is this true of children, resulting in an impaired, over-taxed, nervous organization. There is an unequal development of brain and body. The teeth are prematurely erupted while imperfectly developed. The roots are prematurely absorbed. Their removal causes the premature eruption of the permanent teeth which, being brought into contact with external influences too early, are in their turn subject to early decay. The present system of education is more largely responsible for the vast amount of dental caries than anything else. We place the responsibility of raising our children on school teachers who, in nine cases out of ten, take up the business only as a step to something paying better, and who have no sense of the responsibility involved. A child has no business to be put to hard study till nature has provided it with a perfect apparatus with which to prepare the food to build up brain and body. Do not wean a child till it has an unbroken row of teeth. Do not put it to hard study till it has an unbroken row of permanent teeth. Nature will tell you when to put your child to school and when he is fit for it!—*Items of Interest.*

(*To be continued.*)

